# Systematic review: antihypertensive drug therapy in patients of African and South Asian ethnicity

**DOI:** 10.1007/s11739-016-1422-x

**Published:** 2016-03-30

**Authors:** Lizzy M. Brewster, Gert A. van Montfrans, Glenn P. Oehlers, Yackoob K. Seedat

**Affiliations:** Department of Vascular Medicine, F4-222, Academic Medical Center, University of Amsterdam, Meibergdreef 9, 1105 AZ Amsterdam, The Netherlands; Department of Internal Medicine, Academic Medical Center, University of Amsterdam, Amsterdam, The Netherlands; Department of Cardiology, Academic Hospital of Paramaribo, Paramaribo, Suriname; Nelson R Mandela School of Medicine, Faculty of Health Sciences, University of KwaZulu Natal, Private Bag. 7, Congella, 4013 Durban, South Africa

**Keywords:** Hypertension, Antihypertensive drugs, Systematic review, African continental ancestry group, South Asian, Ethnic groups

## Abstract

Despite the large differences in the epidemiology of hypertension across Europe, treatment strategies are similar for national populations of white European descent. However, hypertensive patients of African or South Asian ethnicity may require ethnic-specific approaches, as these population subgroups tend to have higher blood pressure at an earlier age that is more difficult to control, a higher occurrence of diabetes, and more target organ damage with earlier cardiovascular mortality. Therefore, we systematically reviewed the evidence on antihypertensive drug treatment in South Asian and African ethnicity patients. We used the Cochrane systematic review methodology to retrieve trials in electronic databases including CENTRAL, PubMed, and Embase from their inception through November 2015; and with handsearch. We retrieved 4596 reports that yielded 35 trials with 7 classes of antihypertensive drugs in 25,540 African ethnicity patients. Aside from the well-known blood pressure efficacy of calcium channel blockers and diuretics, with lesser effect of ACE inhibitors and beta-blockers, nebivolol was not more effective than placebo in reducing systolic blood pressure levels. Trials with morbidity and mortality outcomes indicated that lisinopril and losartan-based therapy were associated with a greater incidence of stroke and sudden death. Furthermore, 1581 reports yielded 16 randomized controlled trials with blood pressure outcomes in 1719 South Asian hypertensive patients. In contrast with the studies in African ethnicity patients, there were no significant differences in blood pressure lowering efficacy between drugs, and no trials available with mortality outcomes. In conclusion, in patients of African ethnicity, treatment initiated with ACE inhibitor or angiotensin II receptor blocker monotherapy was associated with adverse cardiovascular outcomes. We found no evidence of different efficacy of antihypertensive drugs in South Asians, but there is a need for trials with morbidity and mortality outcomes. Screening for cardiovascular risk at a younger age, treating hypertension at lower thresholds, and new delivery models to find, treat and follow hypertensives in the community may help reduce the excess cardiovascular mortality in these high-risk groups.

## Background

The increasing ethnic diversity of the European population is likely to bring a greater diversity in disease and disease patterns to the doctor’s office. Around 33 million immigrants live in the European Union. It is estimated that a third of these immigrants are from other European countries, while immigrants from non-European countries are mainly African (25 %, with more than half North-African), or Asian (21 %) [1].

Despite the large differences in the epidemiology of hypertension across Europe [2], treatment strategies tend to be similar for national populations of white European descent. Nevertheless, in particular patients of South Asian and sub-Saharan African descent tend to have more hypertension and diabetes, and more target organ damage and cardiovascular mortality at a younger age than patients of white European descent. In addition, hypertension occurs earlier in life in these patients groups, bringing about a faster progression from normotension to hypertension, with higher mean blood pressures than in white patients [3–22].

While little is reported regarding the pathophysiology of hypertension in South Asian patients, abundant data in patients of African descent indicate there is greater salt sensitivity, blunted nocturnal dipping, and enhanced vasoconstriction in this group [3–8, 10, 12–14, 16–21]. South Asians are genetically diverse, but members of this population subgroup share a high cardiovascular risk, with more severe atherosclerosis reported, and ischaemic end organ damage at a younger age even with lower cholesterol levels than in whites [11]. Thus, hypertension seems to be a more aggressive disease, occurring at a younger age in these patient groups. This could have important implications for hypertension screening and management.

In patients of all ethnicity groups, non-pharmacological intervention to reduce hypertension and cardiovascular risk, including dietary adjustments, physical exercise, weight reduction, smoking cessation, and reduction of excessive alcohol intake should be part of hypertension management. In addition, stress reduction and relaxation exercise might aid in reducing blood pressure [23]. In particular, diets high in potassium and calcium and low in sodium, such as the (DASH) diet, have documented blood pressure lowering efficacy [24–30]. These measures are thought to be effective in hypertensives across ethnic groups, but increasing evidence indicates that the very low salt intake (<1500 mg or <65 mmol sodium per day) recommended for persons of African ethnicity [31–33], has been associated with increased mortality in this group, potentially related to activation of the renin angiotensin system [31, 32]. Even so, high salt intake (>2300 mg or >100 mmol sodium per day) is still considered detrimental to cardiovascular health [32], and moderate salt restriction continues to have a place in the management of hypertension in all ethnic groups.

However, most patients with hypertension will need drug therapy aside life style measures. Therefore, in this paper, we review the evidence on randomized trials of antihypertensive drug treatment in African and South Asian ethnicity patients, and propose practical approaches for the European situation.

## Methods

The participation of patients of ethnic minority groups in major, international clinical trials is generally too low to calculate the primary outcome with sufficient power [34]. Therefore, we systematically reviewed the evidence on the efficacy of antihypertensive drug therapy to reduce blood pressure and morbidity and mortality outcomes, and pooled the existing data.

Systematic searches were conducted in November 2015, with our previous systematic review on patients of African ethnicity [16, 17] updated and expanded. In brief, we used the Cochrane systematic review methodology, [35] and defined a highly sensitive search strategy to retrieve original reports of randomized controlled trials in hypertensive African and South Asian ethnicity patients, providing original quantitative data on the effect of antihypertensive monotherapy on blood pressure (trial duration at least 2 weeks) vs concurrent placebo treatment, or antihypertensive mono or combination therapy on morbidity or mortality outcomes (trial duration at least 1 year).

We included only trials with major drug classes in adults, men and non-pregnant women, with uncomplicated primary hypertension (no history of, or current cardiovascular events or ESRD). Trials that considered oral antihypertensive treatment with thiazide and thiazide-like diuretics, calcium-channel blockers, centrally acting agents, peripheral adrenergic neuron antagonists, angiotensin-converting enzyme (ACE) inhibitors, or angiotensin II receptor blockers were eligible for inclusion.

We conducted separate searches and data analysis for these two ethnic groups. Searches were performed in electronic databases (Embase, PubMed, Cochrane Library CENTRAL, Literatura Latino-Americana y del Caribe en Ciencias de la Salud (LILACS), African Index Medicus, and for South Asian patients, IndMED) from their inception through November 2015, without language restriction.

These databases have different software and therefore different search languages, but a typical search strategy for trials in patients of African ethnicity was, “(Black* OR Afri* OR AFRO* OR Creole OR Carribean OR Caribbean OR negr* OR ethnic* OR blacks) AND (hypertension OR antihypertensive) AND randomized”; and for South Asians: the first step was “(South Asian OR South Asians OR India OR Indian OR Hindustani OR Bangladesh OR Nepal OR Sri Lanka OR Ceylon OR Pakistan)”.

Search yields from all databases were considered and analysed separately to prevent merging errors and to enhance trial retrieval. Furthermore, we contacted experts and performed hand search. We did not include trials in diabetics only, with experimental drugs, or with complementary medicines.

We used data extraction forms to collect trial data. With pilot searches, we retrieved very few placebo controlled trials in South Asians, and decided to review drug vs drug trials in this group. For drug vs drug trials with multiple treatment arms, we followed the Cochrane handbook methodology and combined the comparison groups into one group of “other drugs” [35]. African or South Asian descent (ancestry, or ethnicity) were defined as respectively of sub-Saharan African descent, or Indian subcontinental descent as indicated by the authors. We included only randomized controlled trials, and methodological quality was further assessed using the Jadad score, based on the description of randomization, blinding, and accountability of all patients, including withdrawals in each of the study groups, and the underlying reasons. Subgroups were based on gender and geographical location, and compliance data were assessed in trials with mortality outcomes.

### Statistical analysis

Quantitative analysis of outcomes was based on intention-to-treat results (primary) and per protocol analysis (secondary). We included data from the first part of crossover studies when such data were available; if not, we included the data these studies provided. Our measure of effect for each study was difference in means (in mmHg) for systemic arterial blood pressure (continuous measure) and relative risk (RR) for dichotomous data. In addition, we calculated achievement of target diastolic blood pressure (DBP <90 mmHg, or reduction of ≥10 mmHg, or ≥10 %, as defined by the author) as the weighted mean of placebo-corrected results per drug class, or in South Asians, vs other drug types.

Missing standard deviations were imputed per drug class. We clinically assessed studies for heterogeneity in patient characteristics, interventions, and outcomes, to decide whether studies should be pooled. Furthermore, we used *I*^2^ statistics to quantify the proportion of total variation in the estimates of treatment effect that was due to heterogeneity. We planned to not aggregate results with a high variation across studies (*I*^2^ ≥ 75 %) [17, 35]. When we aggregated studies, we conservatively used the random effects model to estimate the average intervention effect. Data in square brackets are 95 % confidence intervals, unless indicated otherwise. We used Review Manager (RevMan) software, version 5 (Cochrane Collaboration, Oxford, UK) for the analyses.

## Results

### Patients of African ethnicity

**Fig. 1:**
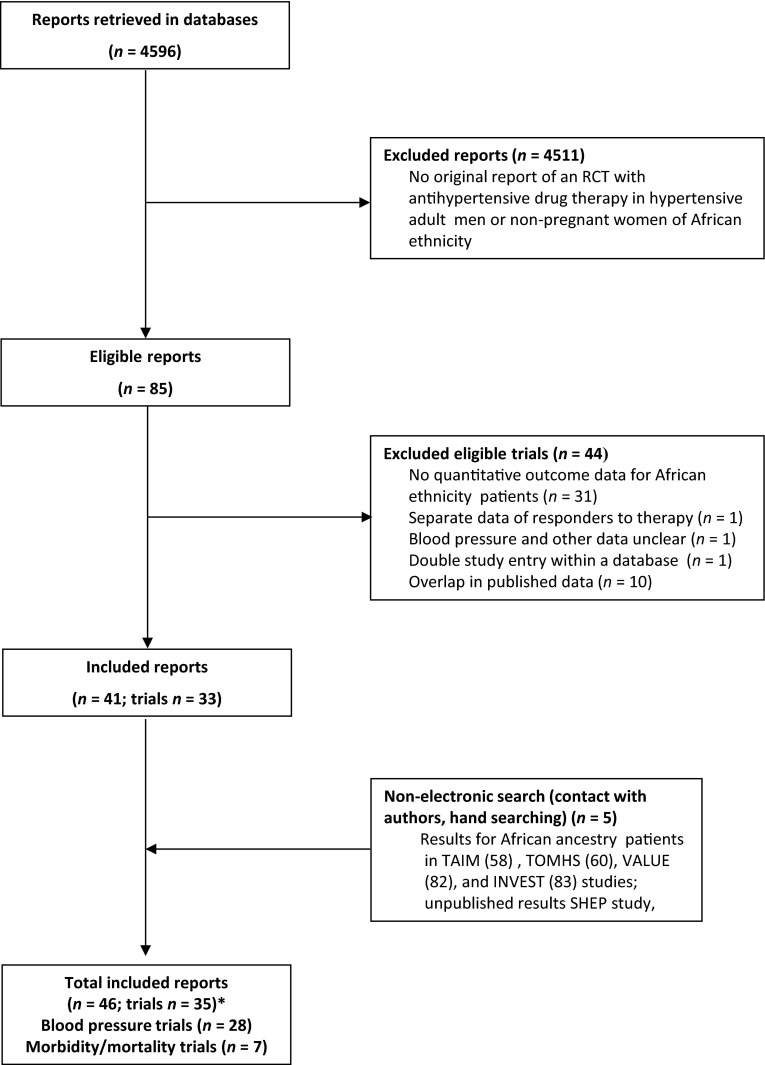
Trial flow: patients of African ethnicity. *Asterisk* with results for African ethnicity patients in the Materson [47, 48], TAIM [57, 58], TOMHS [59, 60], SHEP ([68] and unpublished report), AASK [75, 76, 81] and ALLHAT [79, 80, 86, 88]; LIFE [73, 85, 87] studies contained in more than one report. Most excluded papers were not an RCT; and of the RCT’s retrieved, most were either not an RCT in hypertensives, or an RCT’s in other ethnic groups, an RCTs with combination therapy, drug vs drug trials, or in particular for morbidity and mortality trials, multiple overlapping reports concerning these trials

**Fig. 2:**
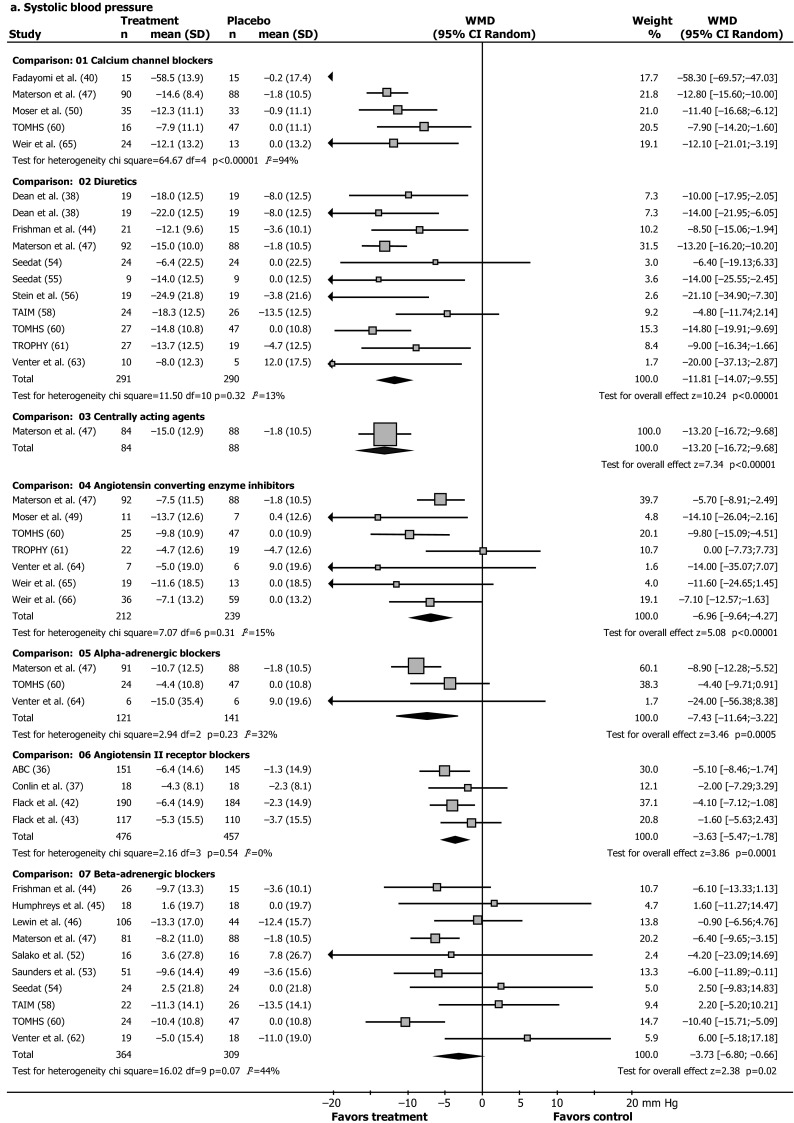

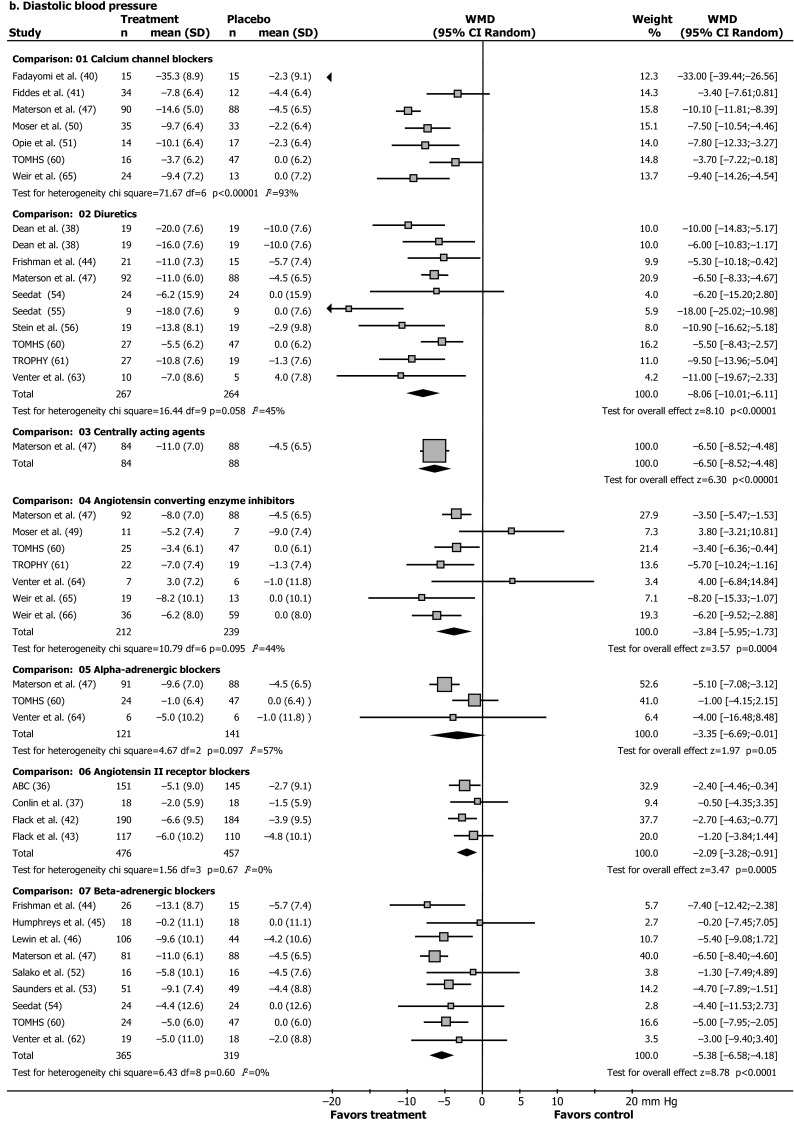
Effect of different antihypertensive drugs on blood pressure in patients of African ethnicity. **a** Systolic blood pressure. **b** Diastolic blood pressure. **a**, **b** Our previous review [17] was updated (November 2015). Except for two nebivolol studies [46, 53], no new trials with single drugs vs placebo and blood pressure outcomes were retrieved. Random, random-effects model. Results are reported as weighted mean differences in reduction of systolic and diastolic blood pressure (mmHg) from baseline to endpoint with the use of different antihypertensive drugs compared to placebo. *Squares* are weighted mean differences in reduction of SBP/DBP (mmHg). The *size of the squares* represents study weight, and *horizontal lines* represent 95 % CIs. *Arrowheads* depict data outside the scale. When a study provided only the placebo-drug difference, we entered a “nil” for placebo results. Results for Materson and colleagues’ study and Weir and colleagues’ study are weighted means of older and younger people and patients receiving a high and a low-salt diet, respectively. *Black diamonds* are pooled estimates. Results for calcium-channel blockers were not pooled because the size of the effect was heterogeneous. *ABC* Association of Black Cardiologists, *TAIM* Trial of Antihypertensive Interventions and Management, *TOMHS* Treatment of Mild Hypertension Study, *TROPHY* Treatment in Obese Patients with Hypertension [36–66]

Full reports or abstracts from 4596 references of papers yielded 35 trials with 7 classes of antihypertensive drugs, in 25,540 patients. Blood pressure was the main outcome measure in 28 of these trials (Figs. 1, 2; Table 1) [36–66], and morbidity or mortality in seven trials (Table 2) [67–88]. Our 2015 update included two new trials with blood pressure outcomes on nebivolol [46, 53], and eight new reports on morbidity and mortality outcomes (five reports with new subgroup analyses from the ALLHAT and LIFE, and AASK trials, and three new reports of the VALUE, INVEST, and ACCOMPLISH trials) [81–88]. Trials were clinically comparable in describing the results of randomized controlled interventions with antihypertensive drugs in African ethnicity patients with hypertension, but the age range, inclusion blood pressure, drugs and drug dose varied (Tables 1, 2). Since we retrieved only two new blood pressure trials considering monotherapy with nebivolol vs placebo, the results of the 2015 update are similar to the data reported previously, as depicted in Fig. 2a, b. As a post hoc outcome, nebivolol was analysed separately as well because of the presumed different mechanism of action [46, 53]. Nebivolol is thought to enhance nitric oxide generation [46, 53]. However, the pooled weighted mean difference in systolic (SBP) and diastolic pressure vs placebo of these two trials is respectively SBP −3.38 mmHg, 95 % CI [−8.38; 1.62]; *I*^2^ 33 %; and DBP −5.00 mmHg, 95 % CI [−7.41; −2.59] (*I*^2^ = 0 %). With the addition of these relatively large trials to the pooled analysis (Fig. 2a) the size of the effect of beta-adrenergic blockers on systolic blood pressure was similar, but the confidence interval became narrower, and statistically significant from placebo [pooled estimate for systolic blood pressure without nebivolol −3.53 [−7.51; 0.45] (*I*^2^ = 50 %) [17], and with nebivolol −3.73 [−6.80; −0.66] (*I*^2^ = 44 %), Fig. 2a].

**Table 1:** Characteristics of studies in African ethnicity patients: blood pressure outcomes

References	Participants of African ethnicity				Drug intervention vs placebo	Treatment duration	Outcome measure (BP)	Analysis of results	Adverse effects	Jadad score					
	*N*	Country	Age (years)	BP (mmHg)	Total daily dose (mg)^a^					RA	MR	DB	MB	DO	Total
ABC [36]	304	USA	Mean 52	DBP 91–105	Candesartan cilexetil 32	8 w	Cont./dichot.	ITT	Reported	1	–	1	1	1	4
Conlin et al. [37]	18^b^	USA	Mean 52	DBP 90–109	Losartan 50^c^	4 w	Cont.	ITT	ND	1	–	1	1	1	4
Dean et al. [38]	60	RSA	Adults	DBP 100–116	Hydrochlorothiazide 50 Mefruside 25	2 w	Cont.	PP	ND	1	–	1	1	–	3
Drayer et al. [39]	58^b^	USA	Mean 53	DBP 95–115	Captopril 200	8 w	Dichot.	PP	ND	1	–	1	–	1	3
Fadayomi et al. [40]	32	Nigeria	Mean 48	DBP >100	Nifedipine 40	6 w	Cont./dichot.	PP	Reported	1	–	1	1	–	3
Fiddes et al. [41]	46	USA	≥55	DBP 95–114	Diltiazem XR 480	8 w	Cont.	ITT	ND	1	–	1	–	–	2
Flack et al. [42]	381	USA	Mean 50	DBP 95–109	Losartan 150	12 w	Cont./dichot.	ITT	Reported	1	–	1	–	1	3
Flack et al. [43]	233^b^	USA/RSA	Mean 52	DBP 95–109	Losartan 50–100	16 w	Cont.	ITT	ND	1	–	1	1	–	3
Frishman et al. [44]	62^b^	USA	≥21	DBP 95–115	Hydrochlorothiazide 25 Bisoprolol 5	4 w	Cont./dichot.	ITT	ND	1	–	1	–	–	2
Humphreys et al. [45]	18	Jamaica	46–63	DBP 100–155	Propranolol 360^c^	2 m	Cont./dichot.	ITT	Reported	1	–	1	1	1	4
Lewin et al. [46]	152	USA	Mean 51	SBP 160–180 DBP 90–100	Nebivolol 20 mg	6 w	Cont./dichot.	ITT	Reported	1	1	1	1	1	5
Materson et al. [47, 48]	621	USA	Mean 58	DBP 95–109	Diltiazem 360 Hydrochlorothiazide 50 Clonidine 0.6 Captopril 100 Prazosin 20 Atenolol 100	8 w/1 y^d^	Cont./dichot.	ITT	ND	1	–	1	–	1	3
Moser et al. [49]	20	Bahamas	32–60	DBP 101–119	Captopril 450	4 w	Cont./dichot.	PP	Reported	1	–	1	–	–	2
Moser et al. [50]	77	USA	26–70	DBP 90–114	Nitrendipine 40	5 w	Cont./dichot.	PP	ND	1	–	1	1	–	3
Opie et al. [51]	31^b^	RSA	18–75	DBP 95–114	Nisoldipine 30	6 w	Cont.	ITT	ND	1	–	1	1	–	3
Salako et al. [52]	20	Nigeria	37–60	DBP 95–120	Alprenol 400^c^	8 w	Cont.	PP	Reported	1	–	1	1	1	4
Saunders [53]	301	USA	Mean 51	DBP 95–109	Nebivolol 40 mg	12 w	Cont.	ITT	Reported	1	–	1	–	1	3
Seedat [54]	24	RSA	Adults	DBP 100–115	Chlorthalidone 100 Atenolol 25^c^	4 w	Cont.	ITT	Reported	1	–	1	–	1	3
Seedat [55]	9	RSA	Mean 44	DBP ≥110	Mefruside 25 Debrisoquine 20^c^	4 w	Cont./dichot.	ITT	ND	1	1	1	–	1	4
Stein et al. [56]	25	Zimbabwe	<70	DPB 96–114	Hydrochlorothiazide 50^c^	6 w	Cont./dichot.	PP	ND	1	–	1	–	1	3
TAIM [57, 58]	98^b^	USA	Mean 46	DBP 90–100	Chlortalidone 25 Atenolol 50^e^	6 m	Cont.	ITT	ND	1	1	1	1	–	4
TOMHS [59, 60]	177	USA	Mean 54	DBP 90–99	Amlodipine 10 Chlortalidone 30 Enalapril 10 Doxasozin 4 Acebutolol 800^f^	1 y	Cont.	PP	Reported for women only	1	–	1	1	–	3
TROPHY [61]	68^g^	USA	21–75	DBP 90–109	Hydrochlorothiazide 50 Lisinopril 40	12 w	Cont.	PP	ND	1	–	1	1	–	3
Venter et al. [62]	50	RSA	25–65	DBP 95–115	Penbutolol 80^g^	12 w	Cont./dichot.	PP	Reported	1	–	1	1	1	4
Venter et al. [63]	15^b^	RSA	25–65	DBP 95–115	Xipamide 20	12 w	Cont.	PP	Reported	1	–	–	–	1	2
Venter et al. [64]	29	RSA	21–65	DBP 95–115	Enalapril 40 Prazosin 20	10 w	Cont./dichot.	PP	Reported	1	–	1	1	1	4
Weir et al. [65]	56^b,h^	USA	Mean 52	DBP 95–115	Isradipine 20 Enalapril 40^i^	4 w	Cont.	PP	ND	1	1	1	1	–	4
Weir et al. [66]	96^b^	USA	Mean 54	DBP 95–114	Trandolapril 16	6 w	Cont.	ITT	Reported	1	–	1	–	1	3

*ABC* Association of Black Cardiologists, *N* number of African ethnicity patients randomized, or evaluated in this review; *USA* United States of America, *RSA* Republic of South Africa, *(D)BP* (diastolic) blood pressure, *mg* milligram, *w* weeks, *m* months, *y* years, *Cont./dichot.* blood pressure reported as continuous or dichotomous outcome, *ITT* intention-to-treat, *PP* per protocol analysis, *ND* no data reported for African ethnicity patients, *RA* randomization, *MR* method of randomization, *DB* double blind, *MB* method of blinding, *DO* dropouts in African ethnicity patients, *TAIM* Trial of Antihypertensive Interventions and Management, *TOMHS* Treatment of Mild Hypertension Study, *TROPHY* Treatment in Obese Patients with Hypertension

^a^Highest daily dose

^b^Number of African ethnicity patients evaluated in this review

^c^Cross-over trial

^d^BP reported as continuous/dichotomous outcome

^e^Other drugs added in 12.5 % of participants

^f^Second drug added in 9.2 % of participants; plus life style interventions

^g^Obese patients

^h^Salt sensitive patients

^i^Plus high/low salt diet

**Table 2:** Trials with morbidity and mortality outcomes in African ethnicity patients

Participants of African ethnicity			Inclusion criteria	Treatment arms^a^	Primary endpoint	Jadad score^b^						Follow up (years)	Primary outcome
Study	*N* (%)	Country				RA	MR	DB	MB	DO	Total		
SHEP	657 (14)	USA	>60 ISH	Chlorthalidone Placebo	Fatal/non-fatal stroke	1	–	1	1	–	3	4.5	NS
LIFE	533 (6)	7 countries^c^	55–80 y LVH	Losartan Atenolol	MI, stroke, CVM	1	1	1	1	–	4	4.8	NS
AASK	1094 (100)	USA	18–70 y GFR 20-65^d^	Ramipril Metoprolol Amlodipine	GFR (usual vs low BP goals)	1	1	1	1	1	5	4.1	NS
ALLHAT	15,094 (35)	USA	>55 y CHD risk	Lisinopril Amlodipine Chlorthalidone Doxazosin	MI + CHD death	1	1	1	1	1	5	4.9	NS
VALUE	639 (4)	31 countries^e^	≥50 y CVD/risk	Valsartan Amlodipine	Time to first cardiac event	1	1	1	1	–	4	4.2	NS
INVEST	3029 (13)	14 countries^e^	>50 y CAD	Atenolol^f^ Verapamil	Death (ACM), MI, or stroke	1	–	–	–	–	1	2.9	NS
ACCOMPLISH	1414 (17)	5 countries^g^	>55 y TOD	Benazepril/HCT Benazepril/Amlodipine	CVD, CVM	1	–	1	–	–	2	3.0	NS

*ISH* isolated systolic hypertension, *LVH* left ventricular hypertrophy, *GFR* glomerular filtration rate, *CAD* coronary artery disease, *CHD* coronary heart disease, *CVD* cardiovascular disease, *CVM* cardiovascular mortality, *TOD* target organ damage, *HCT* hydrochloro-thiazide, *BP* blood pressure, *MI* myocardial infarction, *ACM* all-cause mortality, *NS* no significant difference, *SHEP* the Systolic Hypertension in the Elderly Program [67–69], *LIFE* the Losartan Intervention for Endpoint Reduction in Hypertension Study [70–73, 85, 87], *AASK* African American Study of Kidney Disease and Hypertension [74–76, 81], *ALLHAT* Antihypertensive and Lipid Lowering Treatment to Prevent Heart Attack Trial [77–80, 86, 88], VALUE Valsartan Antihypertensive Long-term Use Evaluation trial [82], *INVEST* the International Verapamil-Trandolapril Study [83], *ACCOMPLISH* Avoiding Cardiovascular Events through Combination Therapy in Patients Living with Systolic Hypertension trial [84]

^a^Parallel treatment arms with initial monotherapy, except SHEP (vs placebo), and ACCOMPLISH (initial combination therapy)

^b^Jadad score: *RA* randomization, *MR* method of randomization, *DB* double blind, *MB* method of blinding, *DO* dropouts in African ethnicity patients

^c^98 % of the African ethnicity patients were from the USA

^d^mL/min/1.73 m^2^

^e^Country of origin African-ethnicity patients not reported

^f^Primary add-on drug trandolapril (verapamil arm) and HCT (atenolol)

^g^African ethnicity patients were from the USA

Achievement of target DBP differed by drug class, calcium-channel blockers 46 % (RR 3.39 [2.35; 4.90]; diuretics 31 % (RR 2.49 [1.68; 3.69]; beta-adrenergic blockers 24 % (RR 1.97 [1.43; 2.72]; centrally acting agents 23 % (RR 2.22 [1.35; 3.63]; angiotensin II receptor blockers 19 % (1.77 [1.41; 2.21]; alpha-blockers 13 % (RR 1.71 [1.02; 2.86]; and ACE inhibitors 10 % (RR 1.35 (0.81; 2.26); with a RR of >1.0 indicating a beneficial effect.

Thus, the aggregated data show a greater effect of calcium blockers and diuretics, while beta-adrenergic blockers and ACE inhibitors are the least effective drugs to lower SBP and DBP, respectively. The cause of these differences in drug responses is largely unknown. Our findings are in accord with the suppressed activity of the renin-angiotensin-aldosterone system in hypertensive patients of African ethnicity, and the high activity of creatine kinase, promoting vasoconstriction and salt retention [8, 16]. As a consequence, patients of African ethnicity are significantly less sensitive to drugs that block the renin-angiotensin-system (angiotensin-converting enzyme inhibitors and angiotensin II receptor blockers) and beta-blockers [16]. Genetic and pharmacokinetic differences do not fully explain these differences [16], but altered cellular functions based on high creatine kinase activity and enhanced phosphoryl group buffer function have been implied in this group, leading to enhanced ATP-dependent responses including greater contractility, salt retention and therapy failure [16, 18], as well as lower NO bioavailability [8, 16].

We predefined subgroups based on gender and on geographical location. However, only 3 small trials out of 28 trials with blood pressure outcomes reported data for men and women (*N* = 146 patients), and this was not further analysed [40, 45, 66]. When we separately analysed US/Caribbean studies, calcium-channel blockers changed SBP by −11.89 mmHg (CI −14.12 to −9.67 mmHg) and beta-blockers led to a change of −4.83 mmHg (CI −7.91 to −1.75 mmHg); the size of the effect of alpha-blockers on DBP became heterogeneous. When we separately analysed data from African studies, however, only calcium-channel blockers remained more effective than placebo for all outcomes analysed. Diuretics did not significantly differ from placebo in achieving the DBP goal (relative risk 3.55 [CI 0.41–31.05]), and ACE inhibitors, beta-blockers, and alpha-blockers did not significantly differ from placebo in reduction of SBP and DBP. None of the African studies used a cutoff baseline DBP of less than 114 mmHg, compared with 7 of the 15 US and Caribbean studies (Table 1). Thus, we could not determine whether the response of African patients truly differed from that of US and Caribbean patients or was rather related to higher baseline blood pressure levels.

We retrieved seven trials with morbidity and mortality outcomes (Table 2) [67–88]. Most included patients were older than 50 years with risk factors for cardiovascular disease, followed for 3–5 years, with cardiovascular events and mortality as main outcome measures. The Jadad scores ranged from 1 to 5 (Table 2). An average of three drugs was needed in an add-on strategy to reach blood pressure goals as defined in the trials. The majority of African descent participants (50–70 %) reached blood pressure control, but 95 % needed combination therapy. In line with the blood pressure lowering efficacy of monotherapy, more patients on calcium blocker-based treatment reached goal blood pressure, while there was a reduced blood pressure lowering response in treatments based on initial monotherapy with angiotensin II receptor blockers or ACE inhibitors [82, 83, 86].

There was no statistical difference between the different treatment arms in primary morbidity and mortality outcomes (Table 2). The main side effects of long-term therapy were newly developed diabetes (diuretics > calcium blockers > ACE inhibitors), and a significantly greater occurrence of cough and angioedema with ACE inhibitors, 72 per 10,000 (0.72 %), vs diuretics (0.04 %), and calcium blockers (0.06 %) for African ethnicity patients in ALLHAT [17, 86].

In the SHEP study, the overall effect of diuretics on the primary outcome stroke in African ethnicity patients was not significantly different from placebo. In subgroup analysis, stroke risk reduced in women of African ethnicity (relative risk 0.36 [CI 0.16; 0.83]) but not in men (relative risk 0.98 [CI 0.39; 2.44]) [69]. However, treatment did reduce cardiovascular events as a secondary outcome (hazard ratio for all cardiovascular events, 0.50 (CI 0.32; 0.78) (unpublished results, SHEP trial investigators).

Furthermore, in the ACCOMPLISH trial, there was no significant difference in African ethnicity patients between the two treatment strategies in retarding the rate of progression of kidney disease, in contrast to patients of other ethnicities where amlodipine/benazepril-based therapy was more effective than hydrochlorothiazide/benazepril [84].

Although ACE inhibitor-based treatment yielded better clinical outcomes in kidney disease in the AASK trial [75], there was no difference in prevention of cardiovascular events by drug type [81], while the results of the ALLHAT trial indicates that cardiovascular morbidity outcomes were worse with treatments based on inhibitors of the renin angiotensin system [86]. The use of lisinopril initiated treatment vs chlorthalidone in patients of African ethnicity was associated with a relative greater risk of morbidity: combined CHD (1.15 [1.02; 1.30]), combined CVD (1.19 [1.09; 1.30]), stroke 1.40 [1.17; 1.68], angina 1.24 [1.07; 1.44]. Heart failure risk was lower with chlorthalidone [86]. No data were provided for lisinopril vs amlodipine.

In line with these findings with ACE inhibitors, the LIFE study showed that losartan-initiated therapy was superior to atenolol-initiated therapy in reducing stroke risk in hypertensive patients of European descent. However, among patients of African descent, losartan-initiated treatment was associated with a nearly significant increase in stroke events compared with atenolol unadjusted hazard ratio, 1.99 [1.00; 3.98] [85], similar to the findings of the primary outcome, a composite outcome including stroke [17, 73]. In addition, the risk for sudden death was 97 % higher in patients of African descent in the LIFE trial, with, at this relatively small sample size (*n* = 533) a trend towards increased risk with losartan [87]. These data indicate that therapy initiated with blockers of the renin-angiotensin-system is associated with a greater cardiovascular morbidity and mortality in patients of African ethnicity.

We defined subgroups based on gender and based on geographical location for morbidity and mortality outcomes. However, morbidity and mortality trials were conducted in the USA only or included only a very small number of non-USA patients (Table 2). The SHEP trial’s outcome for men and women is discussed above, with diuretics not significantly different from placebo in preventing stroke in African ethnicity men. In ALLHAT, men of African descent had the highest absolute stroke risk (mean 6 year rate/100 patients 7.73, 5.90, 5.81, and 5.90, in African ethnicity men, women, and white men, women respectively) and the highest stroke risk with lisinopril of all sex-ethnic groups (6 year rate/100 patients for lisinopril 9.41, 7.25, 5.32, and 5.59, respectively) [88]. Furthermore, pharmacogenetics outcomes differed by gender in the AASK trial, only women randomized to a usual blood pressure goal (mean arterial pressure 102–107 mmHg), and with an A allele at *CYP3A4* A392G, were more likely to reach a target MAP of 107 mmHg [adjusted hazard ratio of AA/AG compared to GG 3.41 (95 % CI 1.20–9.64; *P* = 0.02)]. Among participants randomized to a lower MAP goal, men and women with the C allele at *CYP3A4* T16090C were more likely to reach the target MAP of 107 mmHg [adjusted hazard ratio 2.04 (95 % CI 1.17–3.56; *P* = 0.01)]. In addition, the polymorphisms Arg65Leu, Ala142Val, and Ala486Val of the G protein-coupled receptor kinase gene, *GRK4*, were studied in the AASK Study. Only in men randomized to the usual blood pressure goal (mean arterial pressure 102–107 mmHg), the adjusted “hazard” ratio to reach the goal blood pressure with metoprolol was 1.54 (95 % CI 1.11–2.44; *P* < 0.01) with Ala142Val. There was no association between *GRK4* polymorphisms and blood pressure response to metoprolol in women [16].

Compliance data by ethnicity were only available for the AASK study. Based on self-reported data and pill counts, 23 % of the patients had at least one noncompliant event, non-adherence events (%) per patient year respectively were 7.7, 6.6, and 7.1 for metoprolol, ramipril and amlodipine [74–76].

As approaches to the management of cardiovascular disease risk need to integrate assessment and treatment of several risk factors, we describe the outcome of the lipid lowering treatment arm of the ALLHAT trial (ALLHAT-LLT) [89]. Patients of African ethnicity have been underrepresented in prior trials addressing the effects of cholesterol lowering. Participants treated for hypertension in ALLHAT were eligible for inclusion in ALLHAT-LLT when fasting LDL-C levels were 120–189 mg/dL (3.1–4.9 mmol/L) or 100–129 mg/dL (2.6–3.3 mmol/L) respectively for those with and without known coronary heart disease. The primary outcome was all-cause mortality in patients randomized to pravastatin 20–40 mg vs usual care (respectively *n* = 1769 vs *n* = 1722 African ethnicity patients). Vigorous cholesterol lowering therapy was discouraged in the usual care group, therefore the majority of these patients did not receive lipid lowering drugs (90 % in the second year to 72 % in the sixth year of the trial). There was no difference in the primary outcome of all-cause mortality between pravastatin and usual care (RR for African ethnicity patients 1.01 [0.85–1.19]). In other outcomes, the relative risk for atherosclerotic coronary heart disease events with pravastatin was lower in patients of African descent than in other patients (RR 0.73 [0.58–0.92] vs 1.02 [0.81–1.28]; *P* = 0.03). However, there was a significantly greater stroke risk with pravastatin in patients of African descent (RR 1.12 vs 0.74 in other patients, confidence intervals not reported; *P* = 0.03). As a result, there was no significant effect of pravastatin treatment on combined cardiovascular disease outcomes in hypertensive patients of African ethnicity [89].

### Patients of South Asian ethnicity

**Fig. 3:**
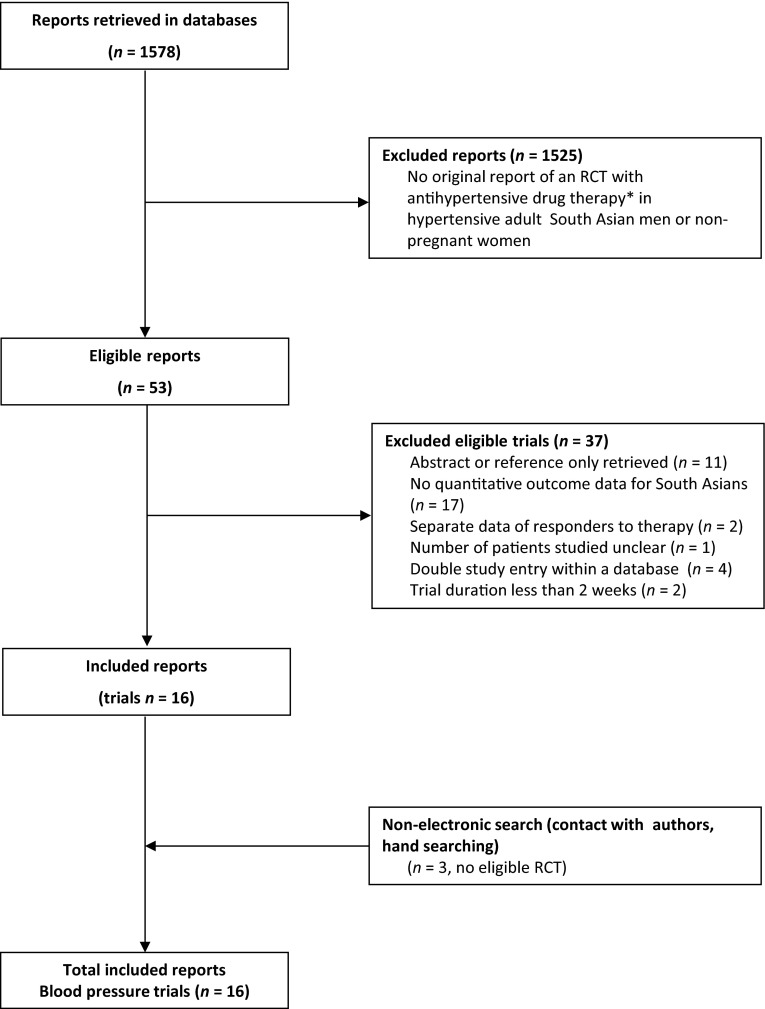
Trial flow: patients of South-Asian ethnicity. *Asterisk* indicate that we included randomized controlled trials (RCT’s) with single drug therapy vs placebo, or vs single drug from another antihypertensive drug class for blood pressure outcomes (at least 2 weeks duration); and with single drug-based or combination therapy for morbidity and mortality outcomes of at least 1 year duration, providing original quantitative data in hypertensive South-Asian adult men or non-pregnant women. Most excluded papers were not an RCT; and of the RCT’s retrieved, most were either not an RCT in hypertensives, or an RCT’s in other ethnic groups, an RCTs with combination therapy, a dose finding trial, trials comparing two drugs within one drug class, or trials of antihypertensive drugs vs non-drug therapy or phytotherapy (*n* = 1525)

With electronic searches (November 2015) we retrieved 1578 papers. We additionally retrieved three trials with hand search, which were not eligible for inclusion. Sixteen randomized controlled trials were included, with blood pressure as the main outcome. Only one trial was placebo controlled, other trials assessed monotherapy with a drug from one drug class vs a drug from another class. We did not include trials that only compared drugs within one antihypertensive drug class. The 16 included trials (Fig. 3; Table 3) were 4 weeks to 9 months duration (median 8 weeks), containing original data of 6 classes of antihypertensive drugs in 1719 South Asian hypertensive patients without a history of, or current cardiovascular events (*n* = 37 diabetics) [55, 90–104].

**Table 3:** Characteristics of Studies in South Asian ethnicity patients: blood pressure outcomes

References	Participants of South-Asian ethnicity				Drug intervention	Treatment duration	Outcome measure (BP)	Analysis of results	Adverse effects	Jadad score^a^					
	*N*	Country	Age (years)	BP	Total daily dose (mg)					RA	MR	DB	MB	DO	Total
Akat [90]	80	IND	18–65	ND	Telmisartan 40 Enalapril 10	12 w	Cont.	PP	Reported	1	–	–	–	–	1
Ali [91]	163	IND	Mean 52	DBP 95–115	Losartan 50 Amlodipine 5	8 w	Cont.	PP	Reported	1	–	–	–	1	2
Bhatia [92]	30	IND	35–65	DBP 90–115	Enalapril 5 Felodipine 5 Prazosin 2	8 w	Cont.	Unclear	Reported	1	–	–	–	–	1
Devi [93]	161	IND	Mean 50	SBP 140–180 DBP 90–114	Metoprolol 50 Amlodipine 5	8 w	Cont./dichot.	ITT	Reported	1	–	–	–	1	2
Goyal [94]	62	IND	Mean 62	SBP 140–179 DBP 90–109	Telmisartan 80 Amlodipine 10	8 w	Cont./dichot.	PP	Reported	1	–	–	–	–	1
Jalal [95]	120^b^	IND	44–63	DBP 90–100	Amlodipine 10 Lisinopril 10	8 w	Cont./dichot.	Unclear	Reported	1	–	–	–	–	1
Jamali [96]	80	PAK	20–70	ND	Candesartan 16 Atenolol 50	90 d	Cont.	PP	Reported	1	–	–	–	1	2
Joglekar [97]	122	IND	30–70	SBP 140–180 DBP 90–110	Prazosin 5 Atenolol 100	4 w	Cont./dichot.	PP	Reported	1	1	–	–	1	3
Misra [98]	110^c^	IND	30–70	SBP 140–180 DBP 90–110	Prazosin 5 Nifedipine 20	8 w	Cont./dichot.	PP	Reported	1	1	–	–	1	3
Nadeesha [99]	120^d^	IND	Mean 45	ND	Amlodipine 5 Atenolol 25 Enalapril 5 HCT 25	8 w	Cont.	PP	ND	1	–	–	–	1	2
Pareek [100]	300	IND	22–81	SBP 140–159 DBP 90–99	Atenolol 25 CTD 6.25 Amlodipine 2.5	4 w	Cont./dichot.	PP	Reported	1	1	–	–	1	3
Satia [101]	65^e^	IND	45–70	DBP 90–110	Atenolol100 Nifedipine 20	9 m	Cont./dichot.	Unclear	ND	1	–	–	–	–	1
Seedat [55]	11	RSA	33–61	DBP ≥110	Debrisoquine 20 Mefruside 25	4 w^g^	Cont./dichot.	PP	Reported	1	1	1	–	1	4
Shobha [102]	145	IND	18–65	DBP 95–110	Losartan 50 Enalapril 5	8 w	Cont./dichot.	PP	Reported	1	–	1	–	1	3
Sumbria [103]	106^f^	IND	Mean 45	SBP ≥140 DBP ≥90	Metoprolol 200 Telmisartan 160	6 m	Cont.	PP	Reported	1	1	–	–	1	3
Sundar [104]	44	IND	35–60	ND	Nifedipine 40 Atenolol 100 Propranolol 80^h^ Captopril 100	4 w^g^	Cont.	PP	Reported	1	–	–	–	–	1

Total daily dose is the maximum dose used

*N* number of patients randomized, *IND* India, *PAK* Pakistan, *RSA* Republic of South Africa, *BP* blood pressure, *SBP* systolic blood pressure at inclusion, *DBP* diastolic blood pressure at inclusion, *ND* no data, *mg* milligram, *d* day, *w* week, *m* month, *HCT* hydrochlorothiazide, *CTD* chlorthalidone, *Cont./dichot.* blood pressure as continuous/dichotomous outcome, *ITT* intention-to-treat, *PP* per protocol analysis

^a^Jadad score: *RA* randomization, *MR* method of randomization, *DB* double blind, *MB* method of blinding, *DO* dropouts

^b^All patients were diagnosed with primary hypertension and microalbuminuria (30–300 mg/24 h), with creatinine clearance >80 mL/min/l.73 m^2^

^c^All patients had an abnormal lipid spectrum

^d^Number of patients in each treatment arm unknown, equal distribution assumed

^e^52 % of the patients had diabetes

^g^Cross-over trial

^f^In the metoprolol treatment arm, 3.6 % had diabetes at baseline vs telmisartan, 2 %

^h^Data of beta-adrenergic blockers were averaged in the comparison of drug class vs drug class [35]

Blood pressure at inclusion was generally between 140 and 180 mmHg systolic, and 90 to 110 mmHg diastolic. Most trials were conducted in India. The methodological quality of the trials was less than in the African patients, with the Jadad scores between 1 and 4 (median 2). No trial had a Jadad score of 5, and only 2 were double blinded. Most trials reported side effects and drop outs, but intention-to-treat analysis was used in only one (Table 3).

There were no significant differences between drug classes in blood pressure-lowering efficacy, as analysed per comparison presented in the trial data [35], (data not shown). Calculation of the blood pressure lowering effect per drug class was hampered by the limited data and heterogeneity that could not be well accounted for (partly due the small number of trials). However, South Asians ethnicity patients represent a population subgroup where the average effect is of clinical relevance. Therefore, we allowed for heterogeneity in an a posteriori analysis, and used the random effects model to calculate the inverse variance-weighted mean blood pressure lowering effect of the different drug classes (Table 4) [35].

**Table 4:** Systolic, diastolic, and target blood pressure by drug class in South Asian patients

Drug class	Systolic BP, mean reduction [CI]	Target SBP (%)	Diastolic BP, mean reduction [CI]	Target DBP (%)
Calcium blockers	−19.08 [−22.75; −15.42]	52–88	−10.81 [−11.58, −10.04]	46–82
Diuretics	−13.58 [−24.40; −2.76]	ND	−9.75 [−16.30; −3.19]	0^a^
ACE-inhibitors	−22.51 [−24.73; −20.29]	ND	−12.78 [−16.61; −8.95]	44
Alpha-blockers	−10.41 [−19.48; −1.34]	39–44	−10.06 [−13.78; −6.35]	0–65^a^
ATII-blockers	−22.63 [−28.55; −16.70]	80	−14.88 [−16.49; −13.27]	59–97
Beta-blockers	−21.11 [−26.44; −15.77]	76	−13.95 [−16.67; −11.23]	74–77

Depicted are inverse-variance weighted means (CI 95 % confidence intervals) of blood pressure reduction (mmHg) per drug type, and range of target blood pressure achievement (%) in South Asian hypertensive patients. Evidence from randomized controlled trials of antihypertensive monotherapy (*n* = 16; [55, 90–104]). Target blood pressure (*n* = 9 trials) [55, 93–95, 97, 98, 100–102] was defined by authors, usually SBP <140 mmHg; DBP <90 mmHg

*Calcium blockers* calcium channel blockers, *ACE-inhibitors* angiotensin converting enzyme inhibitors, *Alpha blockers* alpha-adrenergic blockers, *ATII blockers* angiotensin II receptor blockers, *beta-blockers* beta-adrenergic blockers, *ND* no data

^a^Trials typically had an inclusion baseline DBP <115 mmHg (Table 3). In the only trial with baseline DPB >110, no patient reached diastolic treatment goal with diuretics or alpha blockers [55]. No data were retrieved on centrally acting agents. There was no significant difference in blood pressure lowering effect of different drug types, using comparisons as reported in the trials

Other effects described included that lisinopril reduced micro-albuminuria (−33 vs −10 % in amlodipine) [95], while diuretics and beta-adrenergic blockers were reported to have the well-known metabolic side effects on lipid and glucose metabolism. Non-diuretic, non-beta-adrenergic blocking drugs had a better metabolic profile [97, 99, 101]. There were no separate data provided based on gender, and no trials with morbidity and mortality outcomes.

## Discussion

The WHO Global Monitoring Framework has set a target of 25 % reduction in premature mortality from non-communicable diseases by 2025, including a 25 % reduction in the prevalence of hypertension [105]. Hypertension is the main cause of cardiovascular disease and death across populations worldwide [106], and if the targets are met, premature CVD deaths are projected to be reduced to 5.7 million as a result of a 26 % reduction for men and a 23 % reduction for women [107]. Globally, decreasing the prevalence of hypertension accounts for the largest risk reduction, followed by a reduction in tobacco smoking for men and obesity for women [107]. Since hypertension may differ in age of onset, severity, and response to treatment in different ethnic groups, the increasing ethnic diversity of the European population creates a need for adjusted guidelines to adequately reduce risk factor level in all ethnic groups.

Antihypertensive drugs are the first cardiovascular therapy for which there was wide recognition of differences in clinical efficacy related to ethno-geographical ancestry [16]. Patients of African descent as a group respond better to calcium blockers and diuretics, while the response to β-adrenergic blockade and inhibition of the angiotensin converting enzyme is attenuated [16, 17]. Currently, self-identified ethno-geographic ancestry is the best available predictor of this differential blood pressure lowering response to antihypertensive drugs [16]. As in African patients, South Asians also develop hypertension at an earlier age, with more end organ damage, but there are no known differences in the blood pressure lowering response to antihypertensive drugs, and despite the greater mortality, to our knowledge there are no trials in South Asians with morbidity and mortality outcomes.

The existing evidence provides ample evidence of higher risk of premature cardiovascular mortality in South Asian and African ancestry groups [3–22]. However, to better quantify this risk and develop more effective guidelines, we need to improve risk assessment, and use risk scores validated for ethnic minorities [108, 109]. To this end, we urgently need European morbidity and mortality outcome data for these ethnic groups, as these are likely to differ from the American and Canadian situation, where far higher treatment and control rates for hypertension are reached [18, 110]. Thus, the risk of premature mortality in South Asian and African ancestry groups in Europe is probably underestimated [5]. Although new approaches to estimate risk in these groups have been launched [108, 109], there is still a need for data to support these.

Also, we need data on whether lower thresholds to start treatment and lower therapeutic goal blood pressures need to be applied [111]. The Systolic Blood Pressure Intervention (SPRINT) trial indicates a lower cardiovascular morbidity and mortality within 3 years with a systolic goal blood pressure <120 vs <140 mmHg. However, this difference does not reach statistical significance in the subgroup of African ethnicity patients, with a relatively small sample size and a substantially lower mean age (−5 years) in this subgroup [111]. The International Society for Hypertension in Blacks [112] advises the initiation of treatment in patients of African ethnicity from 135 systolic or 85 mmHg diastolic blood pressure, and similar approaches have been suggested in South Asians [113].

The strength of this work is that we systematically review the available evidence of antihypertensive drug treatment with monotherapy for uncomplicated hypertension, and combination therapy for morbidity and mortality outcomes in hypertensive patients of African and South Asian ethnicity. The aggregated evidence should facilitate guideline development to reduce premature adverse outcomes in these high-risk population subgroups, but many questions remain. We are not well informed regarding the socio-economic circumstances of trial participants, which may have affected treatment failure [18]. Also, the trials are conducted in the USA, Africa and India mainly, and data on European ethnic populations are scarce. In addition, trials rarely report outcomes for men and women separately. Finally, there are no available quantitative data on antihypertensive therapy to reduce morbidity and mortality in South Asians, and newer, non-drug techniques for blood pressure lowering in therapy-resistant hypertension such as renal denervation are of unknown efficacy in South Asians, while in African ethnicity patients there was no significant difference with a sham procedure [114].

However, since there are ample effective drugs available, reducing hypertension and risk of end organ damage in these ethnic groups may predominantly involve different health management strategies. Public health approaches have been suggested to combat hypertension in all ethnic groups, with better models of screening, delivery of care (nurse-based, door-step care), the use of a registry to treat and follow all hypertensives, and initial low dose combination therapy to increase compliance and blood pressure lowering efficacy, while reducing adverse effects [115, 116]. Hypertensive patients of African or South Asian descent should benefit from these more aggressive approaches.

In summary, hypertension in persons of African or South Asian ethnicity occurs more frequently, and is associated with more therapy failure and more severe and earlier end organ damage. European guidelines for cardiovascular risk management should take this high risk into account. Persons of African or South Asian ethnicity need to be screened at a younger age, and treatment should potentially start at lower thresholds with early use of combination therapy and intensive treatment monitoring to reduce the high premature mortality.
